# Modulation of Cell Death by *M. tuberculosis* as a Strategy for Pathogen Survival

**DOI:** 10.1155/2011/678570

**Published:** 2011-01-04

**Authors:** Markos Abebe, Louise Kim, Graham Rook, Abraham Aseffa, Liya Wassie, Martha Zewdie, Alimuddin Zumla, Howard Engers, Peter Andersen, T. Mark Doherty

**Affiliations:** ^1^Armauer Hansen Research Institute, P.O. Box 1005, Addis Ababa, Ethiopia; ^2^The Centre for Infectious Diseases and International Health, Windeyer Institute of Medical Sciences, Royal Free and University College Medical School, London WC1T 4JF, UK; ^3^Department of Infectious Disease Immunology, Statens Serum Institute, Artillerivej 5, København S, 2300 Copenhagen, Denmark

## Abstract

It has been clearly demonstrated that *in vitro*, virulent *M. tuberculosis* can favor necrosis over apoptosis in infected macrophages, and this has been suggested as a mechanism for evading the host immune response. We recently reported that an effect consistent with this hypothesis could be observed in cells from the blood of TB patients, and in this paper, we review what is known about evasion strategies employed by *M. tuberculosis* and in particular consider the possible interaction of the apoptosis-inhibiting effects of *M. tuberculosis* infection with another factor (IL-4) whose expression is thought to play a role in the failure to control *M. tuberculosis* infection. It has been noted that IL-4 may exacerbate TNF-*α*-induced pathology, though the mechanism remains unexplained. Since pathology in TB typically involves inflammatory aggregates around infected cells, where TNF-*α* plays an important role, we predicted that IL-4 would inhibit the ability of cells to remove *M. tuberculosis* by apoptosis of infected cells, through the extrinsic pathway, which is activated by TNF-*α*. Infection of human monocytic cells with mycobacteria *in vitro*, in the presence of IL-4, appears to promote necrosis over apoptosis in infected cells—a finding consistent with its suggested role as a factor in pathology during *M. tuberculosis* infection.

## 1. Introduction

It is generally accepted that tuberculosis (TB) is responsible for 2-3 million deaths and more than 8 million new cases annually [1]. The majority of these occur in developing countries, especially in Sub-Saharan Africa [2], where a substantial proportion of the population (perhaps as much as a third) is thought to be latently infected. Though they are able to control the initial infection, they may later reactivate their disease if they become immunocompromised [3]. Infection with *M. tuberculosis* is associated with an active inflammatory immune response, characterized by elevated expression of both TNF-*α* [4–7] and IFN-*γ* [8–10]. These two cytokines are essential for controlling mycobacterial infections [11–14] but it is clear that in many cases, *M. tuberculosis* is able to survive this inflammatory process. Indeed, *M. tuberculosis* depends on the induction of an inflammatory response and the subsequent tissue damage for cavitation and dissemination via pulmonary disease to new hosts. It is probably for this reason that it expresses multiple molecules on its surface to promote inflammatory responses by the host.

 It is therefore no surprise that *M. tuberculosis* has evolved a number of mechanisms by which it interacts with, and modulates, the host's immune response. In addition to inflammation-promoting molecules [15], *M. tuberculosis* also expresses surface antigens that can induce IL-10 and IL-4, [16–18] that typically have an anti-inflammatory effect. Elevated expression of IL-4 (a cytokine with pleiotropic activity) has been implicated as a potential virulence factor, both for its anti-inflammatory capacity and apparent ability to promote tissue damage in association with TNF-*α* [19]. Higher levels of IL-4 expression also correlate with heightened immune responsiveness to ESAT-6, a proxy marker for infection in TB contacts [20–22] and for bacterial load. Finally, the ratio of IL-4 to IFN-*γ* or the IL-4 antagonistic splice variant, IL-4*δ*2, appears to be correlated with clinical status and in particular, with TB-related pathology [23–26] rather than of infection. 

These studies suggest that IL-4 (alone or together with TNF-*α*) may play a role in tissue destruction and/or cell death during *M. tuberculosis* infection. Since cell death (by apoptosis) is a mechanism by which the host can remove infected cells [27, 28] while minimizing cell death and tissue destruction in adjacent, uninfected cells [29], this has obvious relevance for the control of *M. tuberculosis* infection. Indeed, there is a substantial body of literature suggesting that *M. tuberculosis* can directly interfere with the apoptosis of infected cells *in vitro* [30, 31] and that this appears to be directly related to virulence [32, 33]. In contrast, nonvirulent mycobacteria have a much weaker effect and, being dependant on dose, may even promote apoptosis [30]. 

This question has come under increasing scrutiny in the last few years, and the mechanisms by which *M. tuberculosis* can inhibit apoptosis are being rapidly identified [34]. However, the relative importance of apoptosis as a virulence mechanism *in vivo* and interaction of apoptotic mechanisms with the host cytokine response have until recently been largely unexplored and it is only recently that this area has come into focus [35].

## 2. *M. tuberculosis* and the Generation of Pathology

*M. tuberculosis* normally enters the host though the mucosal surfaces—via the lung after inhalation of exhaled droplets containing bacteria or less frequently through the gut after ingestion of bacteria (e.g., in milk from an infected animal). Although some *M. tuberculosis*-exposed individuals show no signs of infection or T cell memory—having possibly eliminated the pathogen via the innate immune response—the majority of exposed persons display the induction of a rapid inflammatory response. Cytokine and chemokine release triggers the swift accumulation of a variety of immune cells and, with time, the formation of a granuloma, characterized by a relatively small number of infected phagocytes, surrounded by activated monocyte/macrophages and lymphocytes [36]. Traditionally, the granuloma has been thought of as a containment mechanism of the host, but recent work suggests that granulomas are dynamic entities, growing and shrinking as cells are recruited and die [37]. The granuloma may eventually disappear, leaving a small scar or calcification, and the patient's T cells become responsive to *M. tuberculosis*-derived antigens. However, if bacterial replication is not successfully controlled, the granuloma can increase in size and cellularity. The end point of this process is necrosis, and the tissue destruction caused by necrosis, can breach the mucosal surface allowing the granuloma contents to leak into the lumen of the lung or allowing *M. tuberculosis* to escape into the blood vessels of the lung, leading to further dissemination. Destruction of the lumen of the lung—a process referred to as cavitation—gives rise to the prototypic symptom of TB, a persistent cough with blood in the sputum. At this point the patient is infectious, spreading the bacteria by aerosol.

## 3. Inhibition of Early Host Responses

*M. tuberculosis*'s ability to persist within the host is directly linked to the fate of the immune cells which phagocytose it. The macrophage/monocyte thus occupies a pivotal place, as the prototypic host cell for *M. tuberculosis*, and also as the cell responsible for both killing the bacteria directly and priming immune responses by antigen presentation. *M. tuberculosis* interferes with immune activation at virtually every stage. The processes involved in the pathogen's interference with vesicle trafficking and intracellular killing have been well described [38]. The processes involved in large-scale tissue destruction and cell death, however, remain to be mapped out.

Tissue destruction is not mediated directly by *M. tuberculosi*; the bacterium has little or no lytic activity; it is primarily an immunopathological process. Unlike pathogens such as *Leishmania spp*, which can establish chronic infection by evading the host immune response, *M. tuberculosis* actively provokes it. The pathogen expresses a number of molecules that bind to the host's pathogen-associated molecular pattern (PAMP) receptors, such as the Toll-like Receptor (TLR) family [39]. Interestingly, despite *M. tuberculosis*'s long coevolutionary history with humanity, these molecules are largely conserved, even though most of them do not appear to be essential for the pathogen's growth or invasive ability [40, 41]. The simplest explanation is that *M. tuberculosis* depends on the immunopathology that promotes necrosis both for dissemination within the host and for spread to new hosts, but also subverts this response, to allow it to persist in the host. Moreover, the ability of *M. tuberculosis* to rapidly alter its pattern of gene expression in response to stress [42] suggests that the pathogen may do both: in response to the local microenvironment, it may manipulate immune responses so as to favor apoptosis (reducing inflammation, thus allowing persistent infection) or necrosis (promoting tissue destruction, cavitation, and spread to new hosts).

Inhibition of inflammation at early stages may give *M. tuberculosis* a breathing space to initiate a productive infection. It has been suggested that invasion of phagocytes which are not yet activated is important for the bacteria's survival since exposure of macrophages to IFN-*γ* and/or TNF-*α* before—but not after—infection decreases the ability of pathogenic mycobacteria to inhibit phagosome maturation and function [43] at least partially by upregulating the production of reactive oxygen and nitrogen derivatives [44–48]. Mannose derivatives on the pathogen's surface molecules from pathogenic (but not nonpathogenic) mycobacteria inhibit phagocytosis by activated macrophages [49] perhaps targeting the pathogen to cells less prepared to contain it and inhibiting the initiation of inflammatory responses.

It does this in part, by targeting the very mechanisms involved in activating cell-mediated immunity. Though TLR2/4 ligation can initiate the inflammatory cascade in response to mycobacterial infection [50–53], it appears that interference in IFN-*γ*-signaling via TLR signaling is also a potential virulence mechanism [54]. The 19 kDa lipoprotein of *M. tuberculosis* appears to be a virulence factor [55] that reduces overall immunity to the bacterium in mice [56]. It is known to bind to TLR1/2 on host cells [57, 58] with resulting inhibition of inflammatory cytokine production (reducing expression of over a third of the IFN-*γ*-activated genes [59]), and reduced antigen-processing and MHC II expression [59–61]. The virulence factor ESAT-6 has a similar effect, also operating through TLR-2 [62], apparently by modulating TCR signaling pathways downstream of the proximal TCR signaling molecule, ZAP70 [63]. And other factors such as phosphoglycolipids bind to other PAMPs to induce IL-4 and IL-13, apparently contributing to virulence [16, 17, 64], and modulating cytokine expression in concert with other factors [65].

Indeed, *M. tuberculosis* appears to actively modulate cytokine expression at multiple levels. The mannose derivative lipoarabinomannan (LAM), which is expressed by pathogenic (but not nonpathogenic) mycobacteria, can bind to the DC-SIGN molecule, expressed on the surface of dendritic cells. The binding of LAM to DC-SIGN inhibits maturation and induces dendritic cells to secrete IL-10 [18, 66]. This inhibits antigen presentation, expression of MHC molecules, and expression of costimulatory receptors. Consistent with this, recent studies have found that expression of IL-10 is significantly elevated in TB patients with active disease [67–69]. LAM binding to DC-SIGN also inhibits the production of IL-12 by affected antigen-presenting cells. IL-12 is crucial to immunity to *M. tuberculosis*, as indicated by the effect of gene polymorphisms on susceptibility to TB, and the extreme susceptibility to mycobacterial disease of individuals with lesions in genes of the IL-12 and IL-12R pathways [70, 71]. Control of IL-12 expression is key to the expansion and activation of IFN-*γ*—secreting CD4 T cells which are crucial for immunity to TB, as shown by the susceptibility of animals or patients defective in CD4 T cell function or IFN-*γ* expression or recognition [72–76].

## 4. Activating and Modulating the Adaptive Immune to *M. Tuberculosis*

Both CD4 and (to a lesser extent) CD8 T cells are thought to be crucial to containing *M. tuberculosis* infection via IFN-*γ* production and possibly cytotoxicity [77–79]. As discussed above, *M. tuberculosis* appears to subvert the host's immune response, in part by directly countering the activation of T cell—particularly Th1-responses.

Consistent with this, IFN-*γ* recall responses are generally reduced in patients with advanced TB [80], while IL-4 is elevated [81–83]. The level of IL-4 gene expression appears to correlate with both disease severity in TB patients [81, 82] and risk of subsequent disease in TB-exposed individuals [23, 25]. The IFN-*γ*/IL-4 ratio increases in most patients during therapy, but decreases in contacts that become ill, suggesting that this state is directly related to the disease [25]. This is supported by reports that increased production of the IL-4 antagonist IL-4*δ*2 is seen in individuals who are controlling TB in its latent stage [20] and that the IL-4*δ*2 /IL-4 ratio increases during treatment of TB patients [25] and in those TB patients who respond most rapidly to therapy [84]. Similar observations have also been made in animal models of TB [85, 86]. A poor prognosis in TB is associated with a low IFN-*γ*/IL-10 ratio just as is seen for IFN-*γ*/IL-4 [8, 25, 87]. Altering the balance between IFN-*γ* and IL-4 or IL-10 production and function thus seems to be a second major survival strategy for *M. tuberculosis,* and the studies above suggest that when this balance is shifted towards IL-4, the result is increased pathology.

Although IL-4 can inhibit the effect of IFN-*γ* by decreasing the production of IFN-*γ* response factor-1 (IRF-1), a transcriptional element that enhances expression of IFN-*γ*-inducible genes such as iNOS [88], high levels of IL-4 are not associated with an absence of inflammatory factors. The proinflammatory cytokine TNF-*α* is a crucial component for protection against *M. tuberculosis*, as shown by the rapid reactivation of latent *M. tuberculosis* infection in people treated with TNF-*α* receptor antagonists [89, 90] and the susceptibility of TNF-*α*-deficient animals to *M. tuberculosis *[5, 7]. Nonetheless, TNF-*α* mRNA is elevated in TB patients [4] and in TB/HIV-infected patients elevated levels of TNF-*α* were associated with necrosis [91]. It has been suggested that while it is essential for protection, that in the presence of elevated levels of IL-4, TNF-*α* appears to promote tissue damage rather than protection [19, 92], possibly by a cooperative effect of transcription [93, 94]. These studies indicate that *M. tuberculosis* seems to have multiple mechanisms devoted to inhibiting both IFN-*γ* and TNF-*α* function and that the pathogen can evade killing by the immune system while still generating the pathology it needs for dissemination—and suggest that IL-4 may play a crucial role.

## 5. Cytokines, Cell Death, and Pathology

One clue to the mechanisms possibly involved is reports showing that resolving granulomas are rich in apoptotic cells and that inhibition of apoptotic capacity leads to reduced ability to control *M. tuberculosis* [95]. Granulomas are metabolically active sites, with cells being continually recruited and eliminated [37]. This can occur by several processes—but apoptosis or necrosis feature prominently. It has been suggested that apoptosis is a “silent” method whereby the host can remove infected cells [27, 28] while minimizing cell death in adjacent, uninfected cells, thus decreasing tissue destruction [29]. Antigens from engulfed apoptotic cells are presented, thus enabling cross-priming of the immune response [96]. Modeling studies suggest that TNF-*α* is one of the strongest factors controlling monocyte recruitment to the granuloma and that TNF-*α*-driven apoptosis is the strongest negative factor [97]. This is not surprising: TNF-*α* is a potent inducer of cell death by apoptosis [98]. Necrosis, on the other hand, is associated with the lysis of the infected cell, release of viable *M. tuberculosis,* and damage to the surrounding tissue [29] and TNF-*α* is also a major player here [91]. The centre of large unresolved granulomas often becomes necrotic and as mentioned above, this tissue destruction is an essential feature in the spread of *M. tuberculosis*.

There is now a substantial body of evidence from both *in vitro* and *in vivo* studies indicating that virulent *M. tuberculosis *(but not avirulent mycobacteria) can inhibit apoptosis, and that this may represent an escape mechanism whereby the pathogen can avoid the death of its host cell by apoptosis (and the internalized bacteria along with it as the apoptotic cell is digested) [32, 99–105]. Recent work suggests that *M. tuberculosis* can actively promote necrosis over apoptosis, consistent with the idea that this is a survival/virulence mechanism for the bacteria [106–109]. Supporting this hypothesis, studies indicate that elevated levels of necrosis are associated with genetic susceptibility to *M. tuberculosis* in mice [110] or virulence of human-derived clinical isolates [111] and that control of apoptosis via CD43/TNF-*α* inflammatory responses is important for control of *M. tuberculosis* [112]. Some of the genes involved such as *nuoG* have already been identified [113]. 

## 6. Interplay between TNF-*α*, IL-4, and Cell Death *In Vivo*

We therefore have started to examine the significance of TNF-*α*-mediated apoptosis in human TB. Recently published data [4] indicates that there is a strong upregulation of genes for factors that promote apoptosis in PBMC from individuals with active disease, including TNF-*α* and its receptors, *Fas* and *FasL* and pro-Caspase 8, when compared to exposed individuals without active disease. This is consistent with an important role for apoptosis in human TB. The fact that expression of these molecules are elevated in those with overt disease and also that the degree of expression of TNF-*α* correlated with severity of pathology in humans (author's unpublished data and [91, 114]) suggests that TNF-*α* is directly involved in the generation of immunopathology. However, it is hard to reconcile inhibition of apoptosis as a mechanism for pathology if expression of apoptotic genes is highest in those with the worst pathology. A possible explanation for this is the observation that while expression of proapoptotic markers was elevated in PBMC from TB patients, when the CD14+ monocytic fraction was examined, the reverse was true [4]. Our conclusion was that monocytes from TB patients—but not monocytes from those infected with *M. tuberculosis* but asymptomatic, such as individuals with latent TB—were likely less responsive to extrinsic stimuli promoting apoptosis such as TNF-*α*. Further, we hypothesized that since it was highly unlikely that the majority of CD14+ cells in the blood were infected with *M. tuberculosis*, this effect was likely modulated by soluble factors. IL-4 is an obvious candidate, given that it is also the most elevated in these patients, it declines as symptoms abate during treatment [25], and its modulation of necrosis induced by TNF-*α* has been suggested in the past [115, 116]. Increased IL-4 and TNF-*α* expressions are also apparently associated with severity of pathology in mouse model [117], but interestingly, the only study to look at these factors in granulomas from human disease found no association between IL-4 and necrosis—though as the authors note, they could not distinguish between IL-4 and the IL-4 antagonistic splice variant IL4*δ*2 [91] which renders this difficult to interpret.

## 7. Interplay between TNF-*α*, IL-4, and Cell Death* In Vitro*

In the current absence of more data from human studies, we have examined this hypothesized interaction *in vitro*, infecting the human monocytic cell line THP-1 to observe what, if any, effect IL-4 had on the expression of genes that have been shown to be differentially regulated by mycobacterial infection [118–122] particularly those involved in activation of the extrinsic (inflammation-induced) pathway of apoptosis (TNF-*α*, TNFR1, TNFR2, *Fas, FasL*, and Caspase 8). Although not a perfect substitute, THP-1 cells have been frequently used as proxies for alveolar macrophages and have been used in many prior studies of mycobacteria-induced apoptosis [123]. We therefore infected these cells with the virulent *M. tuberculosis* Erdman strain (a clinical isolate) as a prototypical virulent mycobacteria, while the TB vaccine strain BCG Danish 1337 was used as the prototypical avirulent strain. Pilot experiments with the H37Rv and H37Ra virulent and avirulent strains were also done, with similar results to those reported here (data not shown). Bacterial infections were titrated down from a high dose (MOI of 50) down to a low dose (MOI of 5) based on previous publications [124]. We chose the higher dose, based on previous work, which suggested that a higher MOI was needed to induce rapid and detectable apoptosis *in vitro *[30, 123, 125]. 

To ensure that the infection protocol used induced significant levels of apoptosis, we infected THP-1 cells in the presence or absence of IL-4 at 10 ng/ml and monitored apoptosis and necrosis with a cell death ELISA kit, optimized to detect both apoptosis and necrosis. As can be seen in Table 1, after 24 hours, infection with BCG led to a major increase in the amount of apoptosis. Interestingly, the level of apoptosis was slightly reduced by IL-4 treatment alone, though this did not significantly decrease the increase of apoptosis induced by BCG. However, supernatants from the same cultures were assayed for total cell death to assess necrosis and this revealed that IL-4 had a significant effect on the balance of cell death. While IL-4 alone did not significantly affect the level of necrosis, in combination with BCG infection, it had a clear pronecrotic effect. Thus, IL-4 appears to bias cell death slightly towards necrosis over apoptosis, and this effect was enhanced by BCG infection (Table 1).

The results with *M. tuberculosis* Erdman were strikingly different from BCG. At 24 hours, *M. tuberculosis* infection slightly reduced apoptosis and this effect was marginally (but not significantly) augmented by IL-4 (Table 1). But either *M. tuberculosis* infection or IL-4 treatment led to an increased ratio of cell death by necrosis compared to apoptosis.

This effect required living bacteria, as heat killed bacteria had no significant effect on apoptosis or necrosis after 24, 48, or 72 hours of culture (data not shown). We also looked at the effect of BCG and *M. tuberculosis* infection (with or without IL-4) after 72 hours of culture and obtained very similar results (data not shown). Finally, we confirmed the ELISA data by FACS analysis for annexin V, which was expressed by 16.28% after 24 hours of culture with BCG, while only 7.26% of control cells were positive. In the presence of IL-4, only 7.06% of BCG-exposed cells were annexin-V positive, confirming the ELISA data. These data are thus consistent with earlier studies suggesting that virulent (but not avirulent) mycobacteria are capable of inhibiting apoptosis, possibly as a defence mechanism against clearance by the host [32, 33]. In addition, the data suggest that IL-4 can also have a mild antiapoptotic effect—though it appears in this *in vitro* model that this inhibition of apoptosis by IL-4 does not prevent cell death, so much as renders host cells more susceptible to death by necrosis—potentially releasing bacteria which could reinfect adjacent cells, thus further promoting inflammation and immunopathology.

## 8. Effect of IL-4 and Mycobacterial Infection on Expression of Apoptosis-Modulating Genes

To examine the mechanism behind the IL-4 effect, we examined expression of multiple genes involved in activating pathways of induced cell death. It was clear from the apoptosis data (Table 1) that the processes driving apoptosis had already started by 24 hours. We thus performed the PCR analyses after 24 hours of culture, using quantitative PCR to compare the mRNA expression in infected and uninfected cells with or without IL-4 added to the cultures. As shown in Table 1, mycobacterial infection induced a strong TNF-*α* response at 24 hours, and strongly activated expression of the genes for the two TNF-*α* receptors. All of these activating effects were antagonized by IL-4. We also analyzed the supernatants from these cultures and found that in parallel with the induction of TNFR2 mRNA by BCG and *M. tuberculosis*, there was a significant increase (*P* < .01) in the amount of soluble TNFR2 protein detectable in culture supernatants 24 hours after infection (data not shown). This increase was identical for BCG and *M. tuberculosis* and was not inhibited in the presence of IL-4, suggesting that in the presence of IL-4, infected cells continue to shed the TNFR2 receptor at increased levels (compared to uninfected cells), at the same time in which mRNA production is downregulated by IL-4, potentially leading to reduced surface expression and further decreasing the responsiveness of these cells to TNF-*α*. This is consistent with the picture we drew from patient PBMC [4].

 Gene expression for the proapoptotic molecule *Fas* was not affected by BCG infection, although it was significantly decreased by IL-4. In *M. tuberculosis*-infected cells, however, *Fas* expression declined significantly, (Table 1). Since this is likely to render *M. tuberculosis*-infected cells more resistant to Fas-mediated death, we also assessed expression of *FasL* in these cells, to gain an idea of what effect they might have on sensitized cells that came into contact with them. However, despite some variability, no significant differences in *FasL* expression were seen that could be attributed to IL-4 or *M. tuberculosis* infection (Table 1).

Downstream of both *Fas* and the TNF-*α* receptor complexes lies one of the major activating molecules of the extrinsic death pathway, Caspase 8. In BCG-infected THP-1 cells, pro-Caspase 8 transcription increased dramatically and this increase was inhibited by IL-4 consistent with the effects seen on apoptosis. In contrast, in *M. tuberculosis*-infected cells, the opposite was seen, with falling pro-Caspase 8 expression. IL-4 also reduced pro-Caspase 8 expression by itself, but this effect was not significantly different from that induced by *M. tuberculosis* infection. To determine if the decrease in Caspase 8 induced by *M. tuberculosis* infection could be countered by falling levels of apoptosis-antagonising molecules, we also assessed the levels of gene expression for the antiapoptotic molecule FLIPs. Here, however, we found significantly increased expression induced by *M. tuberculosis* infection (*P* < .01), suggesting that if anything, the antiapoptotic effect of decreased Caspase 8 would be amplified. Neither IL-4 nor BCG had a significant effect on FLIPs (data not shown).

In total, these data are consistent with prior findings that *M. tuberculosis* has an apoptosis-blocking effect and indicate that this affects not just the intrinsic pathway but also extrinsic activation of apoptosis mediated through the pro-Caspase 8 molecule, which avirulent mycobacteria do not share. In addition, they suggest that this is potentiated by IL-4, which promotes necrosis instead, supporting a role in the virulence of *M. tuberculosis*. The data also indicate that this antiapoptotic effect occurs at the gene transcription level and affects multiple gene pathways—though the simple experiments presented here are indicative, not definitive.

## 9. A Model for *M. Tuberculosis* Pathogenesis

There is a significant body of evidence from both *in vitro* and *in vivo* studies indicating that virulent *M. tuberculosis* can inhibit apoptosis and that this may represent an escape mechanism whereby the pathogen can avoid the death of its host cell—and the internalized bacteria along with it [32, 99–105]. Knock-in studies using the *nuoG* gene of *M. tuberculosis* showed that this gene conferred the ability to inhibit apoptosis and increased virulence in mice to avirulent mycobacteria, while its deletion rendered *M. tuberculosis* less able to inhibit apoptosis of infected THP-1 cells [113]. A number of genes involved in membrane repair and lipid biosynthesis have also been identified [34]. All of these studies indicate that *M. tuberculosis* actively interferes with the intrinsic pathway of apoptosis in the infected host cell as a means of virulence and that disregulation of the host's lipid metabolism is a major pathway for generating pathology [126] and promoting necrosis over apoptosis [34]. 

The picture for inhibition via the extrinsic apoptotic pathway is also rapidly becoming filled in. Knockout studies of the OppA and OppD genes have implicated the peptide transporters encoded by Rv3665c-Rv3662c and Rv1280c-Rv1283c as inhibitors of apoptosis and this is associated with decreased production of cytokines, including TNF-*α* [127]. Likewise, the hypothetical proteins Rv3654c and Rv3655c appear to interfere with the extrinsic pathway by diminishing the availability of active Caspase 8 through posttranscriptional modification [128]. Inhibition of signaling via members of the TNF receptor superfamily (TNF-*α* and Fas) has long been suggested as a major factor [32, 129, 130] for modulating pathology and more of the genes apparently involved in this process are being identified [131–133]. Interestingly, these findings are tying identified genotypes (such as *nuoG* mutants) to the same mechanisms—production of TNF-*α* and reactive oxygen species—already associated with defence and immunopathology in TB [113, 131, 132].

*M. tuberculosis* infection is known to induce TNF-*α* production, but *in vivo*, infection of host cells does not occur in a vacuum, but in the presence of a variety of immunomodulating factors. We hypothesize that one such factor, IL-4, a cytokine whose expression appears to correlate with a poorer prognosis after *M. tuberculosis* infection [21, 23–25, 92, 134] when combined with TNF-*α*, may worsen TB-related pathology, possibly by biasing cell death towards necrosis instead. If this effect is replicated *in vivo*, (and our data in clinical studies suggest it is [4]) it might help explain why a bias toward IL-4 expression can lead to aggravated pathology in TB [20, 26, 134, 135]. In addition, IL-4 strongly inhibits the expression of the pro-apoptotic molecule TNF-*α* and its two receptors, which are otherwise increased by mycobacterial infection—an effect which may be exacerbated since mycobacterial infection appears to promote the shedding of the soluble form of the receptors [4] that can act as competitive inhibitors. Inhibiting TNF-*α* in primate studies appears to promote pathology [136]. All of this supports the hypothesis that control of apoptosis via CD43/TNF-a inflammatory responses is important for control of *M. tuberculosis* [106, 108, 112]. Finally, IL-4 appears to play a role in the differentiation of M2 (or anti-inflammatory) macrophages, [137–139], which not only promote IL-4 and IL-10 production, but also handle arginine and iron—two important resources for *M. tuberculosis*— differently from M1 macrophages [140, 141]. We suggest expanding the mechanisms by which *M. tuberculosis* actively interferes in this process to suggest that the induction of IL-4, which has been linked to virulence, does so via multiple pathways, and at least partially by promoting cell death by necrosis instead of apoptosis. Identifying the mycobacterial factors which drive this process could offer potential new targets for vaccine and drug development and we are thus investigating *M. tuberculosis* factors that may be involved.

## Figures and Tables

**Table 1:** Alteration in cell death by apoptosis or necrosis in THP-1 cells infected with BCG or *M. tuberculosis* Erdmann (MOI 50) assessed at 24 hours by the Cell Death Detection ELISA^PLUS^ photometric enzyme immunoassay (Roche Diagnostics, Lewes, UK) which measures cell death by both apoptosis and necrosis on fractionated samples. Associated changes in mRNA for the major host genes involved in the activation of the extrinsic pathway of apoptosis were assessed by quantitative Real-Time PCR, using HuPO as a housekeeping gene for normalization. Results shown are relative to untreated cells, of the means of assays from a single experiment (representative of 4) performed in triplicate (ELISA) or quadruplicate (RT-PCR). Values marked in bold text represent a significant increase, those in italics a significant decrease. The ANOVA test (with Dunnett's multiple comparison posttest for all groups against untreated controls) was used for analyses between groups. In all instances, a *P* value <.05 was considered significant. A value of “<.1” indicates below the limit of detection, with “ND” indicates that the experiment was not done.

Infected with:	Fold change over uninfected THP-1 cells								
		Apoptosis	Necrosis	TNF-*α*	TNFR1	TNFR2	Fas	FasL	Caspase 8
null	+ IL-4	*0.67 *±* 0.13 *	*0.79 ± 0.19*	0.51	0.68	0.57	*0.66*	0.96	0.67
BCG	− IL-4	**3.38 ± 0.04**	1.06 ± 0.18	**2.15**	**15.59**	**18.99**	*0.13*	ND	**4.35**
BCG	+ IL-4	**3.29 ± 0.08**	**2.68 ± 0.14**	*0.05*	2.27	3.00	<*0.1 *	ND	0.61
Erdmann	− IL-4	*0.44* ± *0.22 *	1.13 ± 0.30	**17.43**	**4.96**	**5.20**	*0.65*	1.30	*0.36*
Erdmann	+ IL-4	*0.37* ± *0.12 *	**1.34 ± 0.07**	**4.21**	1.38	**1.89**	*0.34*	1.15	*0.35*
